# Supplementary material for: Mapping the evolution of fertility support policies in China: A content and instrumental analysis
Source: PLoS One. 2025 Oct 9;20(10):e0332137. doi: 10.1371/journal.pone.0332137 (PMC12510515; doi:10.1371/journal.pone.0332137)
Supplement: S1 Appendix — (ZIP) [file pone.0332137.s001.zip › S1 Appendix. 226 original policy documents/71-国务院关于落实《政府工作报告》重点工作部门分工的意见(2018)(FBM-CLI-2-313112).doc]

国务院关于落实《政府工作报告》重点工作部门分工的意见(2018)

**发布部门：** [国务院](https://www.pkulaw.com/law/chl?Aggs.IssueDepartment=5)

**发文字号：**国发〔2018〕9号

**发布日期：**2018.04.01

**实施日期：**2018.04.01

**时效性：** [现行有效](https://www.pkulaw.com/law/chl?Aggs.TimelinessDic=01)

**效力级别：** [国务院规范性文件](https://www.pkulaw.com/law/chl?Aggs.EffectivenessDic=XC0203)

**法规类别：** [机关工作综合规定](https://www.pkulaw.com/law/chl?Aggs.Category=00301) [营商环境优化](https://dobusiness.pkulaw.com/)

国务院关于落实《政府工作报告》重点工作部门分工的意见
（国发〔2018〕9号）

各省、自治区、直辖市人民政府，国务院各部委、各直属机构：
　　为全面贯彻党的十九大和十九届二中、三中全会精神，深入落实中央经济工作会议精神和十三届全国人大一次会议通过的《政府工作报告》部署，做好今年政府工作，实现经济社会发展目标任务，现就《政府工作报告》确定的重点工作，提出部门分工意见如下：
　　**一、**落实2018年经济社会发展总体要求和政策取向
　　（一）总体要求和主要预期目标。在以习近平同志为核心的党中央坚强领导下，以马克思列宁主义、毛泽东思想、邓小平理论、“三个代表”重要思想、科学发展观、习近平新时代中国特色社会主义思想为指导，全面深入贯彻党的十九大和十九届二中、三中全会精神，贯彻党的基本理论、基本路线、基本方略，坚持和加强党的全面领导，坚持稳中求进工作总基调，坚持新发展理念，紧扣我国社会主要矛盾变化，按照高质量发展的要求，统筹推进“五位一体”总体布局和协调推进“四个全面”战略布局，坚持以供给侧结构性改革为主线，统筹推进稳增长、促改革、调结构、惠民生、防风险各项工作，大力推进改革开放，创新和完善宏观调控，推动质量变革、效率变革、动力变革，特别在打好防范化解重大风险、精准脱贫、污染防治的攻坚战方面取得扎实进展，引导和稳定预期，加强和改善民生，促进经济社会持续健康发展。国内生产总值增长6.5%左右；居民消费价格涨幅3%左右；城镇新增就业1100万人以上，城镇调查失业率5.5%以内，城镇登记失业率4.5%以内；居民收入增长和经济增长基本同步；进出口稳中向好，国际收支基本平衡；单位国内生产总值能耗下降3%以上，主要污染物排放量继续下降；供给侧结构性改革取得实质性进展，宏观杠杆率保持基本稳定，各类风险有序有效防控。继续创新和完善宏观调控，把握好宏观调控的度，保持宏观政策连续性稳定性，加强财政、货币、产业、区域等政策协调配合。（国家发展改革委牵头，工业和信息化部、财政部、人力资源社会保障部、生态环境部、商务部、人民银行、国务院扶贫办等按职责分工负责）
　　（二）积极的财政政策取向不变，要聚力增效。赤字率拟按2.6%安排，比去年预算低0.4个百分点，财政赤字2.38万亿元，其中中央财政赤字1.55万亿元，地方财政赤字8300亿元。全国财政支出21万亿元，支出规模进一步加大。中央对地方一般性转移支付增长10.9%，增强地方特别是中西部地区财力。优化财政支出结构，提高财政支出的公共性、普惠性，加大对三大攻坚战的支持，更多向创新驱动、“三农”、民生等领域倾斜。当前财政状况出现好转，各级政府仍要坚持过紧日子，执守简朴、力戒浮华，严控一般性支出，把宝贵的资金更多用于为发展增添后劲、为民生雪中送炭。（财政部牵头，国家发展改革委等按职责分工负责）
　　（三）稳健的货币政策保持中性，要松紧适度。管好货币供给总闸门，保持广义货币M_2_、信贷和社会融资规模合理增长，维护流动性合理稳定，提高直接融资特别是股权融资比重。疏通货币政策传导渠道，用好差别化准备金、差异化信贷等政策，引导资金更多投向小微企业、“三农”和贫困地区，更好服务实体经济。（人民银行、国家发展改革委、财政部、中国银行保险监督管理委员会、证监会、国家外汇局等按职责分工负责）

　　**二、**深入推进供给侧结构性改革
　　（四）坚持把发展经济着力点放在实体经济上。继续抓好“三去一降一补”，大力简政减税减费，不断优化营商环境，进一步激发市场主体活力，提升经济发展质量。（国家发展改革委、工业和信息化部、财政部、交通运输部、人民银行、税务总局、国家市场监督管理总局、国家能源局等按职责分工负责）
　　（五）发展壮大新动能。做大做强新兴产业集群，实施大数据发展行动，加强新一代人工智能研发应用，在医疗、养老、教育、文化、体育等多领域推进“互联网＋”。加快发展现代服务业。发展智能产业，拓展智能生活，建设智慧社会。运用新技术、新业态、新模式，大力改造提升传统产业。加强新兴产业统计。（国家发展改革委、工业和信息化部、科技部牵头，教育部、民政部、财政部、人力资源社会保障部、文化和旅游部、国务院国资委、国家卫生健康委员会、国家市场监督管理总局、国家广播电视总局、体育总局、国家统计局、国家文物局、国家中医药局等按职责分工负责）加大网络提速降费力度，实现高速宽带城乡全覆盖，扩大公共场所免费上网范围，明显降低家庭宽带、企业宽带和专线使用费，取消流量“漫游”费，移动网络流量资费年内至少降低30%，让群众和企业切实受益，为数字中国、网络强国建设加油助力。（工业和信息化部牵头，国家发展改革委、财政部、国务院国资委等按职责分工负责）
　　（六）加快制造强国建设。推动集成电路、第五代移动通信、飞机发动机、新能源汽车、新材料等产业发展，实施重大短板装备专项工程，推进智能制造，发展工业互联网平台，创建“中国制造2025”示范区。大幅压减工业生产许可证，强化产品质量监管。（工业和信息化部、国家发展改革委、科技部、财政部、商务部、国务院国资委、国家市场监督管理总局、国家国防科工局、开发银行等按职责分工负责）全面开展质量提升行动，推进与国际先进水平对标达标，弘扬劳模精神和工匠精神，建设知识型、技能型、创新型劳动者大军，来一场中国制造的品质革命。（国家市场监督管理总局牵头，国家发展改革委、教育部、工业和信息化部、司法部、财政部、人力资源社会保障部、商务部、文化和旅游部、国家卫生健康委员会、国务院国资委、国家国防科工局、国家药品监督管理局、国家知识产权局、全国总工会、共青团中央等按职责分工负责）
　　（七）继续破除无效供给。坚持用市场化法治化手段，严格执行环保、质量、安全等法规标准，化解过剩产能、淘汰落后产能。今年再压减钢铁产能3000万吨左右，退出煤炭产能1.5亿吨左右，淘汰关停不达标的30万千瓦以下煤电机组。加大“僵尸企业”破产清算和重整力度，做好职工安置和债务处置。减少无效供给要抓出新成效。（国家发展改革委、工业和信息化部、财政部、人力资源社会保障部、自然资源部、生态环境部、水利部、应急管理部、人民银行、国务院国资委、国家市场监督管理总局、中国银行保险监督管理委员会、国家能源局、国家煤矿安监局、全国总工会等按职责分工负责）
　　（八）深化“放管服”改革。全面实施全国统一的市场准入负面清单制度。在全国推开“证照分离”改革，重点是照后减证，各类证能减尽减、能合则合，企业开办时间再压减一半。大幅缩短商标注册周期。工程建设项目审批时间再压减一半。推进企业投资项目承诺制改革试点。全面实施“双随机、一公开”监管，决不允许假冒伪劣滋生蔓延，决不允许执法者吃拿卡要。深入推进“互联网＋政务服务”，实现政务服务一网通办。推动更多事项在网上办理，必须到现场办的也要力争做到“只进一扇门”、“最多跑一次”，积极推进“一枚印章管审批”，推广“一窗受理、并行办理”。加强政务服务标准化建设，推进办事材料的目录化、标准化、电子化，开展在线填报、在线提交、在线审查。加快推进线上线下融合，统筹服务资源，统一服务标准，切实解决企业群众办事“多跑腿”等问题。加强宣传推广和引导，提高“互联网＋政务服务”的社会认知度和群众认同感。大力推进综合执法机构机制改革，着力解决多头多层重复执法问题。加快政府信息系统互联互通，打通信息孤岛。清理群众和企业办事的各类证明，凡是没有法律法规依据的一律取消。优化营商环境要破障碍、去烦苛、筑坦途，为市场主体添活力，为人民群众增便利。（国务院办公厅、国务院审改办、国家发展改革委、司法部、财政部、住房城乡建设部、交通运输部、商务部、税务总局、国家市场监督管理总局、国家知识产权局等按职责分工负责）
　　（九）进一步减轻企业税负。改革完善增值税制度，按照三档并两档方向调整税率水平，重点降低制造业、交通运输等行业税率，提高小规模纳税人年销售额标准。大幅扩展享受减半征收所得税优惠政策的小微企业范围。大幅提高企业新购入仪器设备税前扣除上限。实施企业境外所得综合抵免政策。扩大物流企业仓储用地税收优惠范围。继续实施企业重组土地增值税、契税等到期优惠政策。全年再为企业和个人减税8000多亿元，促进实体经济转型升级，着力激发市场活力和社会创造力。（财政部、税务总局牵头，工业和信息化部、交通运输部、商务部等按职责分工负责）
　　（十）大幅降低企业非税负担。进一步清理规范行政事业性收费，调低部分政府性基金征收标准。继续阶段性降低企业“五险一金”缴费比例。降低电网环节收费和输配电价格，一般工商业电价平均降低10%。深化收费公路制度改革，降低过路过桥费用。加大中介服务收费清理整顿力度。全年为市场主体减轻非税负担3000多亿元，不合理的坚决取消，过高的坚决降下来，让企业轻装上阵、聚力发展。（财政部、国家发展改革委、工业和信息化部牵头，民政部、人力资源社会保障部、住房城乡建设部、交通运输部、国家市场监督管理总局、国家能源局等按职责分工负责）

　　**三、**加快建设创新型国家
　　（十一）加强国家创新体系建设。强化基础研究、应用基础研究和原始创新，启动一批科技创新重大项目，高标准建设国家实验室。鼓励企业牵头实施重大科技项目，支持科研院所、高校与企业融通创新，加快创新成果转化应用。国家科技投入要向民生领域倾斜，加强雾霾治理研究，推进癌症等重大疾病防治攻关，使科技更好造福人民。（科技部、国家发展改革委、教育部、工业和信息化部、财政部、国务院国资委、生态环境部、国家卫生健康委员会等按职责分工负责）
　　（十二）落实和完善创新激励政策。改革科技管理制度，科研项目绩效评价要加快从重过程向重结果转变。赋予创新团队和领军人才更大的人财物支配权和技术路线决策权。对承担重大科技攻关任务的科研人员，采取灵活的薪酬制度和奖励措施。探索赋予科研人员科技成果所有权或长期使用权。有悖于激励创新的陈规旧章，要抓紧修改废止；有碍于释放创新活力的繁文缛节，要下决心砍掉。（科技部、国家发展改革委、财政部牵头，教育部、人力资源社会保障部、国务院国资委、中科院、工程院、中国科协等按职责分工负责）
　　（十三）促进大众创业、万众创新上水平。提供全方位创新创业服务，推进“双创”示范基地建设，鼓励大企业、高校和科研院所等开放创新资源，发展平台经济、共享经济，形成线上线下结合、产学研用协同、大中小企业融合的创新创业格局，打造“双创”升级版。（国家发展改革委牵头，教育部、科技部、工业和信息化部、财政部、人力资源社会保障部、农业农村部、商务部、国务院国资委、税务总局、国家市场监督管理总局、中国银行保险监督管理委员会、共青团中央、中国科协、全国工商联等按职责分工负责）设立国家融资担保基金，支持优质创新型企业上市融资，将创业投资、天使投资税收优惠政策试点范围扩大到全国。（财政部、国家发展改革委、科技部、工业和信息化部、税务总局、证监会等按职责分工负责）深化人才发展体制改革，推动人力资源自由有序流动，支持企业提高技术工人待遇，加大高技能人才激励，鼓励海外留学人员回国创新创业，拓宽外国人才来华绿色通道。集众智汇众力，跑出中国创新“加速度”。（人力资源社会保障部、科技部、外交部、公安部、国家移民管理局、全国总工会、中国科协等按职责分工负责）

　　**四、**深化基础性关键领域改革
　　（十四）推进国资国企改革。制定出资人监管权责清单。深化国有资本投资、运营公司等改革试点，赋予更多自主权。继续推进国有企业优化重组和央企股份制改革，加快形成有效制衡的法人治理结构和灵活高效的市场化经营机制，持续瘦身健体，提升主业核心竞争力，推动国有资本做强做优做大。积极稳妥推进混合所有制改革。落实向全国人大常委会报告国有资产管理情况的制度。国有企业要通过改革创新，走在高质量发展前列。（国务院国资委、财政部、国家发展改革委牵头，工业和信息化部、司法部、人力资源社会保障部、自然资源部、人民银行、审计署、国家统计局、中国银行保险监督管理委员会、证监会等按职责分工负责）
　　（十五）支持民营企业发展。坚持“两个毫不动摇”，坚持权利平等、机会平等、规则平等，全面落实支持非公有制经济发展的政策措施，认真解决民营企业反映的突出问题，坚决破除各种隐性壁垒。构建亲清新型政商关系，健全企业家参与涉企政策制定机制。激发和保护企业家精神，壮大企业家队伍，增强企业家信心，让民营企业在市场经济浪潮中尽显身手。（国家发展改革委、工业和信息化部、全国工商联牵头，有关部门按职责分工负责）
　　（十六）完善产权制度和要素市场化配置机制。以保护产权、维护契约、统一市场、平等交换、公平竞争为基本导向，完善相关法律法规。对各种侵权行为要依法严肃处理，对产权纠纷申诉案件要依法甄别纠正。让恒产者有恒心，让投资者有信心，让各类产权所有者安心。强化知识产权保护，实行侵权惩罚性赔偿制度。（国家发展改革委、公安部、司法部、国家市场监督管理总局、国家知识产权局等按职责分工负责）加快技术、土地等要素价格市场化改革，深化资源类产品和公共服务价格改革，打破行政垄断，防止市场垄断。要用有力的产权保护、顺畅的要素流动，让市场活力和社会创造力竞相迸发。（国家发展改革委、商务部、国家市场监督管理总局等按职责分工负责）
　　（十七）深化财税体制改革。推进中央与地方财政事权和支出责任划分改革，抓紧制定收入划分改革方案，完善转移支付制度。健全地方税体系，稳妥推进房地产税立法。改革个人所得税。全面实施绩效管理，使财政资金花得其所、用得安全。（财政部牵头，司法部、税务总局等按职责分工负责）
　　（十八）加快金融体制改革。改革完善金融服务体系，支持金融机构扩展普惠金融业务，规范发展地方性中小金融机构，着力解决小微企业融资难、融资贵问题。深化多层次资本市场改革，推动债券、期货市场发展。拓展保险市场的风险保障功能。改革金融监管体制。深化利率汇率市场化改革，保持人民币汇率在合理均衡水平上的基本稳定。（人民银行、国家发展改革委、工业和信息化部、财政部、中国银行保险监督管理委员会、证监会、国家外汇局等按职责分工负责）
　　（十九）推进社会体制改革。深化养老保险制度改革，建立企业职工基本养老保险基金中央调剂制度。（人力资源社会保障部、财政部牵头，国家发展改革委、国务院国资委、证监会、全国总工会等按职责分工负责）深化公立医院综合改革，协调推进医疗价格、人事薪酬、药品流通、医保支付改革，提高医疗卫生服务质量，下大力气解决群众看病就医难题。深入推进教育、文化、体育等改革，充分释放社会领域巨大发展潜力。（国家卫生健康委员会、财政部、国家医疗保障局、国家发展改革委、人力资源社会保障部、商务部、国家市场监督管理总局、国家药品监督管理局、国家中医药局、教育部、文化和旅游部、国家广播电视总局、体育总局、国家文物局等按职责分工负责）
　　（二十）健全生态文明体制。改革完善生态环境管理制度，加强自然生态空间用途管制，推行生态环境损害赔偿制度，完善生态补偿机制，以更加有效的制度保护生态环境。（生态环境部、国家发展改革委、科技部、工业和信息化部、司法部、财政部、自然资源部、住房城乡建设部、水利部、商务部、人民银行、国家市场监督管理总局、国家能源局、国家林业和草原局等按职责分工负责）

　　**五、**坚决打好三大攻坚战
　　（二十一）推动重大风险防范化解取得明显进展。当前我国经济金融风险总体可控，要标本兼治，有效消除风险隐患。严厉打击非法集资、金融诈骗等违法犯罪活动。加快市场化法治化债转股和企业兼并重组。加强金融机构风险内控。强化金融监管统筹协调，健全对影子银行、互联网金融、金融控股公司等监管，进一步完善金融监管、提升监管效能。（人民银行、国家发展改革委、公安部、工业和信息化部、财政部、国务院国资委、中国银行保险监督管理委员会、证监会、国家外汇局等按职责分工负责）防范化解地方政府债务风险。严禁各类违法违规举债、担保等行为。省级政府对本辖区债务负总责，省级以下各级地方政府各负其责，积极稳妥处置存量债务。健全规范的地方政府举债融资机制。今年安排地方专项债券1.35万亿元，比去年增加5500亿元，优先支持在建项目平稳建设，合理扩大专项债券使用范围。（财政部牵头，国家发展改革委、人民银行、审计署、中国银行保险监督管理委员会、证监会等按职责分工负责）
　　（二十二）加大精准脱贫力度。今年再减少农村贫困人口1000万以上，完成易地扶贫搬迁280万人。深入推进产业、教育、健康、生态和文化等扶贫，补齐基础设施和公共服务短板，加强东西部扶贫协作和对口支援，注重扶贫同扶志、扶智相结合，激发脱贫内生动力。强化对深度贫困地区支持，中央财政新增扶贫投入及有关转移支付向深度贫困地区倾斜。对老年人、残疾人、重病患者等特定贫困人口，因户因人落实保障措施。攻坚期内脱贫不脱政策，新产生的贫困人口和返贫人口要及时纳入帮扶。加强扶贫资金整合和绩效管理。开展扶贫领域腐败和作风问题专项治理，改进考核监督方式。坚持现行脱贫标准，确保进度和质量，让脱贫得到群众认可、经得起历史检验。（国务院扶贫办牵头，国家发展改革委、教育部、工业和信息化部、民政部、财政部、人力资源社会保障部、自然资源部、生态环境部、住房城乡建设部、交通运输部、水利部、农业农村部、文化和旅游部、国家卫生健康委员会、人民银行、国家统计局、国家医疗保障局、国家能源局、国家林业和草原局、开发银行、农业发展银行、中国残联等按职责分工负责）
　　（二十三）推进污染防治取得更大成效。巩固蓝天保卫战成果，今年二氧化硫、氮氧化物排放量要下降3%，重点地区细颗粒物（PM_2.5_）浓度继续下降。推动钢铁等行业超低排放改造。提高污染排放标准，实行限期达标。大力发展清洁能源。开展柴油货车、船舶超标排放专项治理，继续淘汰老旧车。深入推进水、土壤污染防治，今年化学需氧量、氨氮排放量要下降2%。实施重点流域和海域综合治理，全面整治黑臭水体。加大污水处理设施建设力度，完善收费政策。加强固体废弃物和垃圾分类处置，严禁“洋垃圾”入境。（生态环境部、国家发展改革委、国家能源局、科技部、工业和信息化部、公安部、财政部、自然资源部、住房城乡建设部、交通运输部、水利部、农业农村部、商务部、海关总署等按职责分工负责）加强生态系统保护和修复，全面划定并严守生态保护红线，完成造林1亿亩以上，耕地轮作休耕试点面积增加到3000万亩，加强地下水保护和修复，扩大湿地保护和恢复范围，深化国家公园体制改革试点。严控填海造地。严格环境执法和问责。要携手行动，建设天蓝、地绿、水清的美丽中国。（生态环境部、国家发展改革委、财政部、自然资源部、住房城乡建设部、水利部、农业农村部、国家林业和草原局等按职责分工负责）

　　**六、**大力实施乡村振兴战略
　　（二十四）健全城乡融合发展体制机制。科学制定规划，依靠改革创新壮大乡村发展新动能。（国家发展改革委、农业农村部牵头，有关部门按职责分工负责）
　　（二十五）推进农业供给侧结构性改革。促进农林牧渔业和种业创新发展，加快建设现代农业产业园和特色农产品优势区。坚持提质导向，稳定和优化粮食生产。加快消化粮食库存。发展农产品加工业。新增高标准农田8000万亩以上、高效节水灌溉面积2000万亩。培育新型经营主体，提高农业科技水平，推进农业机械化全程全面发展，加强面向小农户的社会化服务。鼓励支持返乡农民工、大中专毕业生、科技人员、退役军人和工商企业等从事现代农业建设、发展农村新业态新模式。深入推进“互联网＋农业”，多渠道增加农民收入，促进农村一二三产业融合发展。（农业农村部、科技部、国家发展改革委、工业和信息化部、财政部、人力资源社会保障部、自然资源部、水利部、商务部、退役军人事务部、国家粮食和物资储备局、国家林业和草原局、供销合作总社等按职责分工负责）
　　（二十六）全面深化农村改革。落实第二轮土地承包到期后再延长30年的政策。探索宅基地所有权、资格权、使用权分置改革。改进耕地占补平衡管理办法，建立新增耕地指标、城乡建设用地增减挂钩节余指标跨省域调剂机制，所得收益全部用于脱贫攻坚和支持乡村振兴。深化粮食收储、集体产权、集体林权、国有林区林场、农垦、供销社等改革，使农业农村充满生机活力。（农业农村部、国家发展改革委、财政部、自然资源部、国家粮食和物资储备局、国家林业和草原局、供销合作总社、国务院扶贫办等按职责分工负责）
　　（二十七）推动农村各项事业全面发展。完善农村医疗、教育、文化等公共服务。改善供水、供电、信息等基础设施，新建改建农村公路20万公里。稳步开展农村人居环境整治三年行动，推进“厕所革命”和垃圾收集处理。促进农村移风易俗。健全自治、法治、德治相结合的乡村治理体系。大力培育乡村振兴人才。坚持走中国特色社会主义乡村振兴道路，加快实现农业农村现代化。（农业农村部、国家发展改革委、教育部、工业和信息化部、民政部、司法部、财政部、自然资源部、生态环境部、住房城乡建设部、交通运输部、水利部、文化和旅游部、国家卫生健康委员会、国家能源局、国家林业和草原局等按职责分工负责）

　　**七、**扎实推进区域协调发展战略
　　（二十八）塑造区域发展新格局。完善区域发展政策，推进基本公共服务均等化，逐步缩小城乡区域发展差距，把各地比较优势和潜力充分发挥出来。加强对革命老区、民族地区、边疆地区、贫困地区改革发展的支持。以疏解北京非首都功能为重点推进京津冀协同发展，高起点规划、高标准建设雄安新区。以生态优先、绿色发展为引领推进长江经济带发展。出台实施粤港澳大湾区发展规划纲要，全面推进内地同香港、澳门互利合作。制定西部大开发新的指导意见，落实东北等老工业基地振兴举措，继续推动中部地区崛起，支持东部地区率先发展。加强对资源型地区经济转型发展的支持。壮大海洋经济，坚决维护国家海洋权益。（国家发展改革委牵头，有关部门按职责分工负责）
　　（二十九）提高新型城镇化质量。今年再进城落户1300万人，加快农业转移人口市民化。完善城镇规划，优先发展公共交通，健全菜市场、停车场等便民服务设施，加快无障碍设施建设。有序推进“城中村”、老旧小区改造，完善配套设施，鼓励有条件的加装电梯。加强排涝管网、地下综合管廊、海绵城市等建设。（国家发展改革委、公安部、财政部、人力资源社会保障部、自然资源部、住房城乡建设部、交通运输部、水利部、商务部、国家能源局、中国残联等按职责分工负责）

　　**八、**积极扩大消费和促进有效投资
　　（三十）增强消费对经济发展的基础性作用。推进消费升级，发展消费新业态新模式。将新能源汽车车辆购置税优惠政策再延长三年，全面取消二手车限迁政策。支持社会力量增加医疗、养老、教育、文化、体育等服务供给。创建全域旅游示范区，降低重点国有景区门票价格。推动网购、快递健康发展。（国家发展改革委、教育部、科技部、工业和信息化部、民政部、财政部、生态环境部、住房城乡建设部、交通运输部、商务部、文化和旅游部、国家卫生健康委员会、税务总局、国家广播电视总局、体育总局、国家医疗保障局、国家邮政局、国家文物局等按职责分工负责）对各类侵害消费者权益的行为，要依法惩处、决不姑息。（国家市场监督管理总局牵头，国家发展改革委、工业和信息化部、商务部、国家知识产权局等按职责分工负责）
　　（三十一）发挥投资对优化供给结构的关键性作用。今年要完成铁路投资7320亿元、公路水运投资1.8万亿元左右，水利在建投资规模达到1万亿元。重大基础设施建设继续向中西部地区倾斜。实施新一轮重大技术改造升级工程。中央预算内投资安排5376亿元，比去年增加300亿元。落实鼓励民间投资政策措施，在铁路、民航、油气、电信等领域推出一批有吸引力的项目，使民间资本进得来、能发展。（国家发展改革委、工业和信息化部、财政部、自然资源部、生态环境部、住房城乡建设部、交通运输部、水利部、国务院国资委、国家能源局、国家铁路局、中国民航局、中国铁路总公司等按职责分工负责）

　　**九、**推动形成全面开放新格局
　　（三十二）推进“一带一路”国际合作。坚持共商共建共享，落实“一带一路”国际合作高峰论坛成果。推动国际大通道建设，深化沿线大通关合作。扩大国际产能合作，带动中国制造和中国服务走出去。优化对外投资结构。加大西部、内陆和沿边开放力度，提高边境跨境经济合作区发展水平，拓展开放合作新空间。（国家发展改革委、外交部、商务部牵头，科技部、工业和信息化部、财政部、自然资源部、交通运输部、人民银行、国务院国资委、海关总署、国家市场监督管理总局、国家国防科工局、国家外汇局、中国铁路总公司等按职责分工负责）
　　（三十三）促进外商投资稳定增长。进一步拓展开放范围和层次，完善开放结构布局和体制机制，以高水平开放推动高质量发展。加强与国际通行经贸规则对接，建设国际一流营商环境。全面放开一般制造业，扩大电信、医疗、教育、养老、新能源汽车等领域开放。有序开放银行卡清算等市场，放开外资保险经纪公司经营范围限制，放宽或取消银行、证券、基金管理、期货、金融资产管理公司等外资股比限制，统一中外资银行市场准入标准。进一步调整缩减外商投资准入负面清单。加快制定外资基础性法律。全面实行准入前国民待遇加负面清单管理制度。实施境外投资者境内利润再投资递延纳税。简化外资企业设立程序，商务备案与工商登记“一口办理”。（国家发展改革委、教育部、工业和信息化部、民政部、司法部、财政部、商务部、国家卫生健康委员会、人民银行、税务总局、国家市场监督管理总局、中国银行保险监督管理委员会、证监会、国家外汇局等按职责分工负责）全面复制推广自贸试验区经验，探索建设自由贸易港，打造改革开放新高地。（商务部牵头，国务院自由贸易试验区工作部际联席会议成员单位按职责分工负责）
　　（三十四）巩固外贸稳中向好势头。扩大出口信用保险覆盖面，整体通关时间再压缩三分之一。改革服务贸易发展机制。培育贸易新业态新模式。推动加工贸易向中西部梯度转移。积极扩大进口，办好首届中国国际进口博览会，下调汽车进口关税，对部分市场热销日用消费品及药品，较大幅度降低进口税率，抗癌药品力争降到零税率。以更大力度的市场开放，促进产业升级和贸易平衡发展，为消费者提供更多选择。（商务部牵头，国家发展改革委、工业和信息化部、财政部、交通运输部、人民银行、海关总署、税务总局、国家外汇局等按职责分工负责）
　　（三十五）促进贸易和投资自由化便利化。坚定不移推进经济全球化，维护自由贸易，同有关方推动多边贸易谈判进程，早日结束区域全面经济伙伴关系协定谈判，加快亚太自贸区和东亚经济共同体建设。主张通过平等协商解决贸易争端，反对贸易保护主义，坚决捍卫自身合法权益。（商务部牵头，外交部、国家发展改革委、工业和信息化部、财政部、人民银行、海关总署、国家外汇局等按职责分工负责）

　　**十、**提高保障和改善民生水平
　　（三十六）着力促进就业创业。加强全方位公共就业服务，大规模开展职业技能培训，进一步拓展就业岗位，运用“互联网＋”发展新就业形态。今年高校毕业生820多万人，要促进多渠道就业，支持以创业带动就业。扎实做好退役军人安置、管理和保障工作。加大对残疾人等就业困难人员援助力度，确保零就业家庭动态清零。扩大农民工就业，全面治理拖欠工资问题。健全劳动关系协商机制，消除性别和身份歧视，使更加公平、更加充分的就业成为我国发展的突出亮点。（人力资源社会保障部牵头，国家发展改革委、教育部、科技部、工业和信息化部、公安部、民政部、财政部、住房城乡建设部、农业农村部、退役军人事务部、人民银行、国务院国资委、税务总局、国家市场监督管理总局、国家统计局、全国总工会、共青团中央、全国妇联、中国残联、全国工商联等按职责分工负责）
　　（三十七）稳步提高居民收入水平。继续提高退休人员基本养老金和城乡居民基础养老金。合理调整社会最低工资标准。完善机关事业单位工资和津补贴制度，向艰苦地区、特殊岗位倾斜。（人力资源社会保障部、财政部、国家发展改革委等按职责分工负责）提高个人所得税起征点，增加子女教育、大病医疗等专项费用扣除，合理减负，鼓励人民群众通过劳动增加收入、迈向富裕。（财政部、税务总局牵头，国家发展改革委、教育部、司法部、人力资源社会保障部、国家卫生健康委员会、国家医疗保障局等按职责分工负责）
　　（三十八）发展公平而有质量的教育。推动城乡义务教育一体化发展，教育投入继续向困难地区和薄弱环节倾斜。切实降低农村学生辍学率，抓紧消除城镇“大班额”，着力解决中小学生课外负担重问题。多渠道增加学前教育资源供给，重视对幼儿教师的关心和培养，运用互联网等信息化手段对儿童托育中育儿过程加强监管，让家长放心安心。大力发展职业教育，支持和规范社会力量举办职业教育。推进普及高中阶段教育。以经济社会发展需要为导向，优化高等教育结构，加快“双一流”建设，支持中西部建设有特色、高水平大学。继续实施农村和贫困地区专项招生计划。发展民族教育、特殊教育、继续教育和网络教育。加强师资队伍和师德师风建设。加快推进教育现代化，办好人民满意的教育，让每个人都有平等机会通过教育改变自身命运、成就人生梦想。（教育部牵头，国家发展改革委、工业和信息化部、国家民委、财政部、人力资源社会保障部、国家卫生健康委员会、国务院扶贫办、中国残联等按职责分工负责）
　　（三十九）实施健康中国战略。提高基本医保和大病保险保障水平，居民基本医保人均财政补助标准再增加40元，一半用于大病保险，让更多的人能够享受大病保险救助，扩大大病保险病种。扩大跨省异地就医直接结算范围，把基层医院和外出农民工、外来就业创业人员等全部纳入。（财政部、国家医疗保障局、国家卫生健康委员会、中国银行保险监督管理委员会等按职责分工负责）加大医护人员培养力度，加强全科医生、儿科医生队伍建设，推进分级诊疗和家庭医生签约服务。通过发展“互联网＋医疗”、医联体等，把优质医疗资源下沉。继续提高基本公共卫生服务经费人均财政补助标准。坚持预防为主，加强重大疾病防控。改善妇幼保健服务。支持中医药事业传承创新发展。鼓励中西医结合。对境外已上市销售的药品，研究简化进口使用的审批手续，更好地保障群众用药可及。（国家卫生健康委员会牵头，国家发展改革委、工业和信息化部、财政部、教育部、国家医疗保障局、国家药品监督管理局、国家中医药局等按职责分工负责）创新食品药品监管方式，注重用互联网、大数据等提升监管效能，加快实现全程留痕、信息可追溯，让问题产品无处藏身、不法制售者难逃法网，让消费者买得放心、吃得安全。（国家市场监督管理总局牵头，国家发展改革委、公安部、财政部、农业农村部、商务部、国家卫生健康委员会、国务院食品安全办、国家粮食和物资储备局、国家药品监督管理局等按职责分工负责）做好北京冬奥会、冬残奥会筹办工作，多渠道增加全民健身场所和设施。（体育总局、中国残联、国家发展改革委等按职责分工负责）
　　（四十）更好解决群众住房问题。启动新的三年棚改攻坚计划，今年开工580万套。加大公租房保障力度，对低收入住房困难家庭应保尽保，将符合条件的新就业无房职工、外来务工人员纳入保障范围。坚持房子是用来住的、不是用来炒的定位，落实地方主体责任，继续实行差别化调控，建立健全长效机制，促进房地产市场平稳健康发展。支持居民自住购房需求，培育住房租赁市场，发展共有产权住房。加快建立多主体供给、多渠道保障、租购并举的住房制度，让广大人民群众早日实现安居宜居。（住房城乡建设部牵头，国家发展改革委、财政部、自然资源部、农业农村部、人民银行、国务院国资委、税务总局、国家市场监督管理总局、国家林业和草原局、中国银行保险监督管理委员会、证监会、开发银行、农业发展银行等按职责分工负责）
　　（四十一）强化民生兜底保障。稳步提高城乡低保、社会救助、抚恤优待等标准。积极应对人口老龄化，发展居家、社区和互助式养老，推进医养结合，提高养老院服务质量。关心帮助农村留守儿童。加强城乡困境儿童保障。做好伤残军人和军烈属优抚工作。加强残疾人康复服务。健全社会救助体系，支持公益慈善事业发展。倾情倾力做好托底工作，让每一个身处困境者都能得到社会的关爱和温暖。（民政部、国家发展改革委、教育部、财政部、人力资源社会保障部、住房城乡建设部、国家卫生健康委员会、退役军人事务部、国家医疗保障局、全国总工会、中国残联等按职责分工负责）
　　（四十二）打造共建共治共享社会治理格局。完善基层群众自治制度，加强社区治理。发挥好工会、共青团、妇联等群团组织作用。促进社会组织、专业社会工作、志愿服务健康发展。营造尊重妇女、关爱儿童、尊敬老人、爱护残疾人的良好风尚。（民政部、国家发展改革委、教育部、国家民委、公安部、司法部、财政部、人力资源社会保障部、住房城乡建设部、农业农村部、文化和旅游部、国家卫生健康委员会、国务院扶贫办、国务院妇儿工委办公室、全国总工会、共青团中央、全国妇联、中国残联等按职责分工负责）加强社会信用体系建设。（国家发展改革委、人民银行牵头，有关部门按职责分工负责）完善公共法律服务体系，落实普法责任制。推进平安中国建设，严密防范和坚决打击暴力恐怖活动，依法开展扫黑除恶专项斗争，惩治盗抢骗黄赌毒等违法犯罪活动，整治电信网络诈骗、侵犯公民个人信息、网络传销等突出问题，维护国家安全和公共安全。（公安部、安全部、司法部、国家发展改革委、工业和信息化部、财政部、国家市场监督管理总局、国家信访局等按职责分工负责）
　　（四十三）创新信访工作方式。完善社会矛盾多元化解机制。依法及时解决群众合理诉求。深化信访工作制度改革，认真落实《关于进一步加强信访法治化建设的意见》，不断提高信访工作专业化、法治化、信息化水平。全力打好信访矛盾化解攻坚战，有效解决信访突出问题、化解重大风险隐患。深入推进领导干部接访下访，健全完善社会力量参与信访工作机制，切实把信访问题解决在初始、化解在属地。（国家信访局牵头，教育部、公安部、民政部、司法部、人力资源社会保障部、自然资源部、生态环境部、住房城乡建设部、交通运输部、农业农村部、国家卫生健康委员会、退役军人事务部、中国银行保险监督管理委员会等按职责分工负责）
　　（四十四）坚持不懈抓好安全生产。统筹推进安全生产领域改革发展，严格落实安全生产责任，强化安全法治规范约束作用，加强安全风险管控和隐患排查治理，坚决防范遏制重特大事故。（应急管理部牵头，有关部门按职责分工负责）
　　（四十五）提高防灾减灾救灾能力。做好地震、气象、地质等工作。推进防灾减灾救灾体制机制改革。强化自然灾害监测预警和应急处置，加强防灾减灾工程建设，完善防灾减灾救灾社会动员机制。统筹做好灾后恢复重建工作。（应急管理部牵头，有关部门按职责分工负责）
　　（四十六）健全应急管理机制。推进突发事件应急体系建设“十三五”规划实施，加快预警信息发布系统和航空医学救援体系建设，强化综合应急保障能力。加强国际应急支援能力建设，提高国际应急支援水平。推动应急管理标准化建设，健全应急预案体系。加快应急产业发展，推进基层应急管理能力建设和应急管理科普宣教开展，提升社会协同应对能力。（应急管理部、外交部、国家发展改革委、科技部、工业和信息化部、水利部、商务部、文化和旅游部、国家卫生健康委员会、国家市场监督管理总局、国家国际发展合作署、中国气象局、国家粮食和物资储备局等按职责分工负责）
　　（四十七）为人民过上美好生活提供丰富精神食粮。弘扬中华优秀传统文化，继承革命文化，发展社会主义先进文化，培育和践行社会主义核心价值观。加强思想道德建设和群众性精神文明创建。加快构建中国特色哲学社会科学，繁荣文艺创作，发展新闻出版、广播影视、档案等事业。加强文物保护利用和文化遗产保护传承。建好新型智库。加强互联网内容建设。深入实施文化惠民工程，培育新型文化业态，加快文化产业发展。倡导全民阅读，建设学习型社会。深化中外人文交流，增强中华文化影响力。要以中国特色社会主义文化的繁荣兴盛，凝聚起实现民族复兴的磅礴精神力量。（文化和旅游部、外交部、国家发展改革委、教育部、财政部、国家广播电视总局、社科院、国务院发展研究中心、国家文物局等按职责分工负责）

　　**十一、**全面加强政府自身建设
　　（四十八）全面推进依宪施政、依法行政。严格遵守[宪法](https://www.pkulaw.com/chl/7c7e81f43957c58bbdfb.html)法律，加快建设法治政府，把政府活动全面纳入法治轨道。坚持严格规范公正文明执法，有权不可任性，用权必受监督。各级政府要依法接受同级人大及其常委会的监督，自觉接受人民政协的民主监督，主动接受社会和舆论监督，认真听取人大代表、政协委员意见，听取民主党派、工商联、无党派人士和各人民团体意见。政府要信守承诺，决不能“新官不理旧账”。推行政府法律顾问制度。坚持科学、民主、依法决策，凡涉及公众利益的重大事项，都要深入听取各方意见包括批评意见。（各部门按职责分工负责）全面推进政务公开。建立健全例行新闻发布制度，充分利用新媒体传播优势，深入解读阐释政策，加强政务舆情处置回应，更好引导社会预期。加快修订出台《[中华人民共和国政府信息公开条例](https://www.pkulaw.com/chl/117226c0d8725a33bdfb.html)》，做好组织实施工作。进一步推进基层政务公开标准化规范化试点并完成总结验收，提升基层政务公开和政务服务水平。全面推动国务院部门制定主动公开基本目录。（国务院办公厅、司法部、国家市场监督管理总局、国务院新闻办等按职责分工负责）
　　（四十九）全面加强党风廉政建设。推进“两学一做”学习教育常态化制度化，认真开展“不忘初心、牢记使命”主题教育。坚决贯彻落实党中央八项规定及实施细则精神，驰而不息整治“四风”，特别是力戒形式主义、官僚主义。加强审计监督。巩固发展反腐败斗争压倒性态势，把权力关进制度的笼子，坚决惩治各类腐败行为。政府工作人员要廉洁修身，勤勉尽责，自觉接受法律监督、监察监督和人民的监督，干干净净为人民做事，决不辜负人民公仆的称号。（各部门按职责分工负责）
　　（五十）全面提高政府效能。深入推进政府职能转变。优化政府机构设置和职能配置，深化机构改革，形成职责明确、依法行政的政府治理体系，增强政府公信力和执行力。各级政府及其工作人员要提高政治素质和工作本领，求真务实，干字当头，决不能表态多调门高、行动少落实差，决不允许占着位子不干事。要完善激励约束、容错纠错机制，旗帜鲜明给积极干事者撑腰鼓劲，对庸政懒政者严肃问责。（国务院办公厅、司法部、人力资源社会保障部等按职责分工负责）

　　**十二、**做好民族、宗教、侨务、国防、港澳台、外交工作
　　（五十一）全面贯彻党的民族政策。坚持和完善民族区域自治制度。继续加强对民族地区、人口较少民族发展的支持。组织好广西壮族自治区、宁夏回族自治区成立60周年庆祝活动。加强各民族交往交流交融，让中华民族共同体的根基更加坚实、纽带更加牢固。（国家民委牵头，国家发展改革委、教育部、财政部、国务院扶贫办等按职责分工负责）
　　（五十二）全面贯彻党的宗教工作基本方针。坚持我国宗教的中国化方向，促进宗教关系健康和谐，发挥宗教界人士和信教群众在促进经济社会发展中的积极作用。（有关部门按职责分工负责）
　　（五十三）认真落实侨务政策。维护海外侨胞和归侨侨眷合法权益，为他们在国家现代化建设中发挥独特优势和重要作用创造更好条件，激励海内外中华儿女同心奋斗、共创辉煌。（有关部门按职责分工负责）
　　（五十四）继续推进国防和军队改革。以党在新时代的强军目标为引领，牢固确立习近平强军思想在国防和军队建设中的指导地位，坚定不移走中国特色强军之路，全面推进练兵备战工作，坚决有力维护国家主权、安全、发展利益。坚持党对军队绝对领导的根本原则和制度，全面深入贯彻军委主席负责制。建设强大稳固的现代边海空防。完善国防动员体系，加强全民国防教育。维护军人军属合法权益，让军人成为全社会尊崇的职业。深入实施军民融合发展战略，深化国防科技工业改革。各级政府要采取更有力的举措，支持国防和军队建设改革，深入开展拥军优属、拥政爱民活动，使军政军民团结始终坚如磐石、始终根深叶茂。（国家发展改革委、科技部、工业和信息化部、公安部、财政部、交通运输部、退役军人事务部、国家国防科工局、全国拥军优属拥政爱民工作领导小组等按职责分工负责）
　　（五十五）继续全面准确贯彻“一国两制”方针。严格依照[宪法](https://www.pkulaw.com/chl/7c7e81f43957c58bbdfb.html)和基本法办事。全力支持香港、澳门特别行政区政府和行政长官依法施政，大力发展经济、持续改善民生、有序推进民主、促进社会和谐。支持香港、澳门融入国家发展大局，深化内地与港澳地区交流合作。（国务院港澳办牵头，外交部、国家发展改革委、教育部、科技部、财政部、商务部、人民银行、国务院国资委等按职责分工负责）
　　（五十六）继续贯彻对台工作大政方针。坚持一个中国原则，在“九二共识”基础上推动两岸关系和平发展，推进祖国和平统一进程。坚决维护国家主权和领土完整，绝不容忍任何“***”分裂图谋和行径。扩大两岸经济文化交流合作，逐步为台湾同胞在大陆学习、创业、就业、生活提供与大陆同胞同等待遇。（有关部门按职责分工负责）
　　（五十七）推动构建新型国际关系。始终不渝走和平发展道路，积极参与改革完善全球治理，致力于建设开放型世界经济。推进大国协调合作，深化同周边国家睦邻友好和共同发展，加强同发展中国家团结合作。办好博鳌亚洲论坛年会、上海合作组织峰会、中非合作论坛峰会等主场外交。继续为解决国际和地区热点问题发挥负责任大国作用。加强和完善海外利益安全保障体系。与各国一道，为推动构建人类命运共同体不懈努力。（外交部牵头，国家发展改革委、财政部、商务部、人民银行、国家国际发展合作署等按职责分工负责）
　　今年是全面贯彻党的十九大精神的开局之年，是改革开放40周年，是决胜全面建成小康社会、实施“十三五”规划承上启下的关键一年。各地区、各部门要更加紧密地团结在以习近平同志为核心的党中央周围，高举中国特色社会主义伟大旗帜，以习近平新时代中国特色社会主义思想为指导，全面深入贯彻党的十九大和十九届二中、三中全会精神，牢固树立“四个意识”，坚定“四个自信”，依法履职，勤勉尽责，落实好《政府工作报告》重点工作分工，确保圆满完成今年经济社会发展主要目标任务，兑现政府对人民的承诺。一是加强组织领导。各部门要按照分工要求，一把手亲自抓、负总责，抓紧制定推进重点工作的实施方案，于4月20日前报国务院。要细化分解任务，建立工作台账，明确时间表、路线图，严格执行工作责任制，做到有措施、有目标、有责任人。二是积极协调配合。各有关部门在机构改革期间，要做到思想不乱、工作不断、队伍不散、干劲不减，确保工作不出现空档期。坚持“先立后破、不立不破”的原则，新的机构成立后，在其职责调整到位之前，各有关部门要继续按照原职责规定做好各项工作；职责调整到位后，要依据新的职责及时交接，做好重点工作。对涉及跨领域、多部门参与的工作，牵头部门要发挥主导作用，积极沟通协调，协办部门要密切配合，共同推动，切实履职尽责，提高工作针对性和实效性。三是强化督查落实。各部门要坚持说实话、谋实事、出实招、求实效，加强对各地区的指导帮助，做好日常跟踪督办、年中重点抽查和年底总结考核，及时推动解决突出问题，以钉钉子精神做实做细做好各项工作。各省（区、市）人民政府要加强与国务院有关部门工作对接，围绕《政府工作报告》重点工作，结合本地区实际进一步明确工作重点，抓好督查落实。国务院办公厅要加强对各地区、各部门督查工作的督查，建立督查工作总台账，定期做好对账，结合组织开展国务院大督查和专项督查加强跟踪督办，对真抓实干成效明显的要强化表扬激励，对庸政懒政怠政者要严肃追责问责，更好发挥督查鼓励先进、督促后进的作用，切实推动重点工作落实到位。

国务院
2018年4月1日

© 北大法宝：（[www.pkulaw.com](https://www.pkulaw.com/)) 专业提供法律信息、法学知识和法律软件领域各类解决方案。北大法宝为您提供丰富的参考资料，正式引用法规条文时请与标准文本核对。
欢迎查看所有[产品和服务](https://www.pkulaw.com/helps/2.html)。法宝快讯：[如何快速找到您需要的检索结果？](https://www.pkulaw.com/helps/70.html)[法宝](https://www.pkulaw.com/helps/69.html)V6有何新特色？

扫描二维码阅读原文

**原文链接：**<https://www.pkulaw.com/chl/fd21b2dd2f42b4cfbdfb.html>
